# Trends in upper respiratory infection burden among children and adolescents in East Asia, 1990 to 2023, with projections to 2035: An observational study based on the Global Burden of Disease 2023

**DOI:** 10.1097/MD.0000000000049372

**Published:** 2026-06-19

**Authors:** Dong Chen, Botong Sun, Yujie Qian, Jing Ma, Lei Zhang, Dan Qian, Zhi Xu

**Affiliations:** a Department of Pediatrics, Zhangjiagang Fifth People’s Hospital, Zhangjiagang, Jiangsu, China; b Department of Pediatrics, Xinhua Community Health Service Center, Changning, Shanghai, China; c Department of Respiratory and Critical Care Medicine, Zhangjiagang Fifth People’s Hospital, Zhangjiagang, Jiangsu, China; d Science and Education Department, Zhangjiagang Fifth People’s Hospital, Zhangjiagang, Jiangsu, China.

**Keywords:** children and adolescents, disease burden, East Asia, upper respiratory infections (URI)

## Abstract

To evaluate temporal trends in upper respiratory infection (URI) burden among children and adolescents in East Asia from 1990 to 2023 and project trajectories through 2035 using Global Burden of Disease 2023 data. URI incidence, mortality, and disability-adjusted life years (DALYs) for ages 5 to 19 years were extracted for China, DPR of Korea, Japan, Mongolia, Republic of Korea, and globally. Age-standardized rates (age-standardized incidence rate, age-standardized mortality rate, and age-standardized DALYs rate [ASDR]) and estimated annual percentage change were used to assess temporal trends. The socio-demographic index evaluated burden relative to development. Decomposition, age-period-cohort, and Bayesian APC (BAPC) analyses quantified drivers and projected trends through 2035. In 2023, Japan had the highest age-standardized incidence rate (253,071.04 per 100,000) and Mongolia the lowest (105,719.25 per 100,000). Age-standardized mortality rate declined significantly across all countries, most markedly in China (EAPC = −10.97). Burden was greatest at ages 5 to 9 and slightly higher in males. Japan and Republic of Korea showed ASDR substantially exceeding socio-demographic index -expected levels (eff_diff = 43.09 and 37.31). Globally, absolute DALYs increased, driven by population growth and age structure change, partially offset by epidemiological improvement; in East Asian countries, epidemiological improvement was the primary driver of DALYs reduction. Cohort analysis showed generational risk reduction, with a transient period-effect upturn in China during 2019 to 2023. BAPC projections indicate continued global ASDR decline through 2035, rising trends in Japan and Democratic People’s Republic of Korea, and a rebound followed by a plateau in China. URI burden among East Asian children and adolescents has broadly declined but with marked country heterogeneity; Japan and Republic of Korea bear a disproportionately high burden relative to their development level, highlighting the need for targeted prevention strategies.

## 1. Introduction

Upper respiratory infections (URIs) are among the most prevalent acute infectious diseases worldwide, encompassing a broad spectrum of conditions including the common cold, acute pharyngitis, acute tonsillitis, and acute sinusitis, with causative pathogens spanning viruses, bacteria, and atypical organisms.^[1]^ URIs carry an exceptionally high disease burden among children and adolescents, representing one of the leading causes of outpatient visits, school absenteeism, and antibiotic prescriptions in this age group,^[2]^ and imposing a substantial economic burden on families and society.^[3]^ Although the direct mortality attributable to URIs is relatively low, associated complications, including otitis media, acute tracheobronchitis, and pneumonia, remain an important contributor to morbidity among children and adolescents, with lasting adverse effects on academic performance and quality of life, particularly in settings with limited healthcare resources.^[4]^

East Asia is characterized by high population density, diverse climatic conditions, and rapid ongoing urbanization, all of which create favorable conditions for URI transmission. The 5 major countries in the region – China, the Democratic People’s Republic of Korea (DPR of Korea), Japan, Mongolia, and the Republic of Korea – differ markedly in their levels of economic development, healthcare system capacity, and demographic structure, factors that collectively shape the regional pattern of URI disease burden.^[5]^ In recent decades, sustained economic growth and improvements in public health infrastructure across East Asia have contributed to a notable decline in URI-related mortality; nevertheless, the absolute burden of URI remains considerable, driven by population growth, accelerating aging, and the dense human contact associated with rapid urbanization.^[6]^ Furthermore, the non-pharmaceutical interventions implemented during the COVID-19 pandemic (including mask-wearing and physical distancing) transiently suppressed URI transmission, while a post-pandemic rebound in respiratory infections has since been observed and reported across multiple East Asian countries.^[7]^

The Global Burden of Disease (GBD) study represents the most comprehensive framework for systematically quantifying disease burden at global and regional levels and has been widely applied to trend analyses of both communicable and noncommunicable diseases.^[8,9]^ Prior studies drawing on GBD data have predominantly focused on adult populations or all-age URI burden,^[10]^ leaving a relative paucity of systematic analyses targeting the specific demographic of children and adolescents in East Asia. Children and adolescents are at a critical stage of physiological development, with incompletely matured immune systems and high-density congregate settings such as schools and childcare facilities, making the transmission dynamics and disease impact of URIs fundamentally distinct from those observed in adults, an epidemiological specificity that warrants dedicated investigation.^[11]^ In light of this, the present study draws on GBD 2023 data to systematically characterize temporal trends in URI disease burden among children and adolescents in East Asia from 1990 to 2023, and to project future trajectories through 2035, with the aim of providing an evidence base to inform URI prevention and control strategies and guide the allocation of health resources across the region.

## 2. Materials and methods

### 2.1. Data source

Data for this study were obtained from the GBD 2023 study, a comprehensive dataset covering 369 diseases and injuries across 204 countries and territories, publicly accessible through the Global Health Data Exchange online platform (https://vizhub.healthdata.org/gbd-results/). In the GBD 2023 framework, upper respiratory infections are defined as acute infections involving the upper respiratory tract, including the common cold (acute nasopharyngitis), acute pharyngitis, acute tonsillitis, acute laryngitis, acute tracheitis, acute laryngopharyngitis, and acute sinusitis, classified under ICD-10 codes J00 to J06.9 and J36, and ICD-9 codes 460 to 465.9 and 475. Otitis media (ICD-10: H65–H71.93; ICD-9: 381–383.9) is modeled separately in GBD and was excluded from this analysis, though it is recognized as a common complication of URI.^[1]^ We extracted annual data on the incidence, mortality, and disability-adjusted life years (DALYs) of URI, along with their age-standardized rates (ASRs) – including the age-standardized incidence rate (ASIR), age-standardized mortality rate (ASMR), and age-standardized DALYs rate (ASDR) – for the period 1990 to 2023, both globally and for 5 East Asian countries: China, DPR of Korea, Japan, Mongolia, and the Republic of Korea. The study population comprised children and adolescents aged 5 to 19 years, stratified into 3 age groups: 5 to 9, 10 to 14, and 15 to 19 years. The socio-demographic index (SDI) is a composite measure used in the GBD framework to assess the overall socioeconomic development level of different countries and regions.^[8]^ It is derived from 3 dimensions – income per capita, educational attainment, and total fertility rate – with values ranging from 0 to 1, where higher values indicate greater levels of development. In this study, temporal trajectories of SDI against ASDR from 1990 to 2023 were plotted for each country and compared against the global expected frontier to evaluate whether the observed URI burden in each country was commensurate with its corresponding level of socioeconomic development. The frontier difference (eff_diff) was defined as the difference between the observed ASDR and the expected minimum ASDR at a given SDI level; a positive value indicates that the actual burden exceeds the expected level.^[12,13]^

### 2.2. Statistical analysis

For each country and globally, we calculated the absolute numbers and age-standardized rates of URI incidence, mortality, and DALYs. Age-standardized rates were used to eliminate the confounding effect of differences in age structures across regions, thereby enabling meaningful comparisons of disease burden. All estimates are reported with 95% uncertainty intervals for GBD data and 95% confidence intervals (CIs) for estimated annual percentage change (EAPC) estimates. To quantitatively assess long-term temporal trends in URI burden from 1990 to 2023, we calculated the EAPC for ASIR, ASMR, and ASDR. The EAPC was derived by fitting a linear regression model to the natural logarithm of the age-standardized rate against calendar year^[14]^:


$$\begin{array}{c} ln\left(y\right)=\;\alpha +\;\beta \;\times \;year+\;\varepsilon \end{array}$$ (1)

$$\begin{array}{c} \begin{array}{c} EAPC=\left(e^{\beta }-1\right)\times 100\% \end{array} \end{array}$$ (2)

where *y* denotes the age-standardized rate in a given year, α is the intercept, β is the regression coefficient representing the annual rate of change, and ε is the error term. An EAPC > 0 indicates an upward trend, while an EAPC < 0 indicates a downward trend. All results are reported with 95% CIs. Statistical analyses were performed using R software (version 4.4.2; R Foundation for Statistical Computing, Vienna, Austria). While the COVID-19 pandemic (2020–2023) likely disrupted URI epidemiology, we did not exclude these years from primary analyses; instead, joinpoint regression (Section 2.3) was specifically employed to identify pandemic-related inflection points and quantify period-specific changes.

Additionally, temporal trajectories of SDI against ASDR from 1990 to 2023 were plotted for each country and compared against the global expected frontier to assess whether the observed URI burden in each country was commensurate with its level of socioeconomic development.^[15]^

### 2.3. Joinpoint regression analysis

To capture potential nonlinear temporal patterns and pandemic-related fluctuations that a single EAPC may obscure, a piecewise (segmented) log-linear regression – conceptually analogous to joinpoint regression – was additionally applied to the ASIR from 1990 to 2023. The model fits piecewise log-linear segments connected at unknown breakpoints and estimates a segment-specific annual percentage change:^[16]^:


$$\begin{array}{c} \begin{array}{c} ln\left(y\right)=\;\beta_{0}\;+\beta_{1}\;\cdot \;year\;+\;\overset{K}{\underset{k=1}{\sum }}\delta_{k}\;\;\;{\left(year\;\;\;-\tau_{k}\right)}_{+}\;+\;\varepsilon \end{array} \end{array}$$ (3)

where τ_*k*_ denotes the *k*-th joinpoint. Breakpoint locations were estimated by the iterative algorithm of Muggeo, the optimal number of breakpoints was selected using the Bayesian information criterion (BIC), and the significance of each segment-specific annual percentage change was tested via Davies’ test with 95% CIs derived by the delta method. Analyses were performed in R (version 4.4.2) using the segmented package (version 2.2-1), and annual percentage changes with 95% CIs were derived for each segment.

### 2.4. Decomposition analysis

A decomposition analysis was conducted to disentangle the contributions of 3 distinct drivers to the absolute change in URI-related DALYs between 1990 and 2023: population growth; age structure change (shifts in age composition within the 5- to 19-year population, not population aging in the conventional demographic sense); and epidemiological change (i.e., change in the age-standardized rate). Analyses were stratified by sex (male, female, and combined) to identify sex-specific differences in the drivers of changing disease burden.^[17]^

### 2.5. Age-period-cohort (APC) model analysis

The age-period-cohort (APC) model is an advanced analytical framework designed to simultaneously quantify the independent contributions of age, calendar period, and birth cohort effects on disease burden, while distinguishing between net drift (overall trend) and local drift (period-specific trends).^[18]^ In this study, the APC model was applied to examine temporal patterns in URI DALY rates among children and adolescents globally and across the 5 East Asian countries. Model parameters were estimated using the APC web-based tool provided by the National Cancer Institute, with the intrinsic estimator method employed to address the inherent collinearity among age, period, and cohort (defined as age = period − birth year).^[19]^ The intrinsic estimator method imposes a zero-sum constraint on second differences of parameters to resolve the non-identifiability problem, yielding estimable functions (local drifts, curvatures) without making arbitrary assumptions about linear trends.^[19]^ The model assumes log-linear effects and multiplicative relationships between age, period, and cohort. Age effects are expressed as rates per 100,000 person-years; period and cohort effects are expressed as rate ratios relative to the respective reference period or birth cohort, with 95% CIs derived directly from the model.

### 2.6. Bayesian age-period-cohort (BAPC) model for projection

A Bayesian age-period-cohort (BAPC) model was employed to project future URI ASDR trends from 2024 to 2035, based on observed data from 1990 to 2023, for all 6 regions (global and 5 East Asian countries). Building upon the classical APC framework, the BAPC model incorporates Bayesian prior distributions and uses the integrated nested Laplace approximation package for approximate Bayesian inference. Random walk priors of order 2 were applied to age, period, and cohort effects to allow smoothing while preserving flexibility. Model fit was assessed using the Watanabe-Akaike information criterion, and convergence diagnostics confirmed stable parameter estimation. Prediction uncertainty is quantified by 95% prediction intervals (PIs).^[20]^

### 2.7. Ethics

The Institutional Review Board granted an exemption for this study because it utilized publicly accessible, de-identified aggregate data that did not include any confidential or personally identifiable patient information.

## 3. Results

### 3.1. Geographic distribution and temporal trends of URI burden among children and adolescents in East Asia

**Figure 1.**
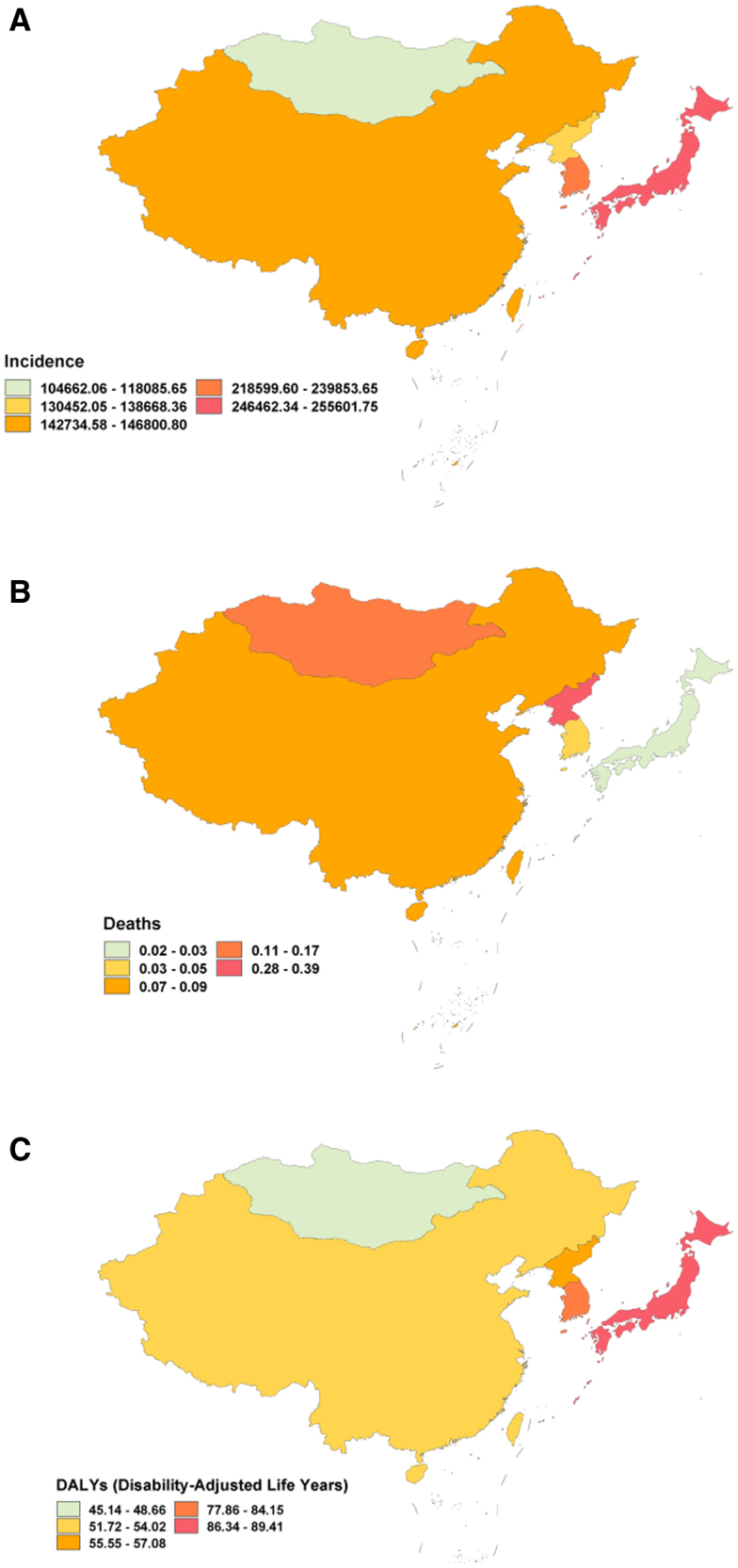
Geographic distribution of age-standardized rates of upper respiratory infections among children and adolescents in East Asian countries in 2023. (A) Age-standardized incidence rate (ASIR, per 100,000 population). (B) Age-standardized mortality rate (ASMR, per 100,000 population). (C) Age-standardized DALYs rate (ASDR, per 100,000 population). ASDR = age-standardized DALYs rate, ASIR = age-standardized incidence rate, ASMR = age-standardized mortality rate, DALYs = disability-adjusted life years.

In 2023, GBD-modeled estimates of URI burden varied substantially across the 5 East Asian countries (Fig. 1, Tables 1–3), though differences may partly reflect data quality and surveillance capacity heterogeneity rather than solely epidemiological factors. Japan had the highest ASIR (253,071.04 per 100,000) and Mongolia the lowest (105,719.25 per 100,000); China, the DPR of Korea, and Mongolia all fell below the global level (168,590.70 per 100,000), whereas Japan and the Republic of Korea exceeded it. From 1990 to 2023, the global ASIR declined gradually (EAPC = −0.24), while rates in China, the DPR of Korea, and Mongolia remained essentially stable (EAPC ≈ 0) (Table 1). ASMR was at or below the global level (0.38 per 100,000) in all 5 countries, with the DPR of Korea matching the global level and the remaining 4 countries recording lower values, with Japan recording the lowest rate (0.02 per 100,000). All regions showed significant declines over the study period, most notably in China (EAPC = −10.97) and Japan (EAPC = −10.35) (Table 2). For ASDR, Japan recorded the highest rate in 2023 (88.52 per 100,000) and Mongolia the lowest (45.59 per 100,000). Declining trends were observed in all countries, with China showing the greatest reduction (EAPC = −3.00) and Japan the smallest (EAPC = −0.32) (Table 3).

**Table 1:** Age-standardized incidence rate (ASIR) of upper respiratory infections and its temporal trends from 1990 to 2023.

Country	ASIR per 100,000 population (95% UI)		EAPCs (95% CI)
	1990	2023	
Global	178,611.83 (161,688.18–198,693.48)	168,590.70 (151,977.32–187,970.90)	−0.24 (−0.26 to −0.21)
China	147,634.12 (131,843.90–165,580.60)	146,800.80 (131,034.41–164,744.08)	0.01 (−0.00 to 0.02)
DPR of Korea	137,066.13 (122,548.04–153,477.22)	136,635.25 (121,851.34–154,490.87)	0.01 (0.00–0.02)
Japan	258,877.12 (234,472.54–291,561.31)	253,071.04 (228,493.36–283,679.29)	−0.07 (−0.08 to −0.06)
Mongolia	105,328.88 (93,422.07–117,303.15)	105,719.25 (92,727.16–118,613.52)	0.00 (−0.00 to 0.01)
Republic of Korea	241,455.76 (217,069.58–272,930.04)	236,549.30 (211,762.84–264,656.49)	−0.06 (−0.07 to −0.06)

ASIR = age-standardized incidence rate, CI = confidence interval, DPR = Democratic People’s Republic, EAPCs = estimated annual percentage changes, UI = uncertainty interval.

**Table 2:** Age-standardized mortality rate (ASMR) of upper respiratory infections and its temporal trends from 1990 to 2023.

Country	ASMR per 100,000 population (95% UI)		EAPCs (95% CI)
	1990	2023	
Global	0.91 (0.20–1.97)	0.38 (0.08–1.09)	−2.94 (−3.10 to −2.78)
China	2.24 (0.33–4.16)	0.09 (0.04–0.28)	−10.97 (−11.64 to −10.29)
DPR of Korea	0.78 (0.17–2.61)	0.38 (0.02–2.61)	−2.21 (−2.41 to −2.00)
Japan	0.49 (0.38–0.61)	0.02 (0.01–0.02)	−10.35 (−11.20 to −9.50)
Mongolia	0.67 (0.12–1.64)	0.12 (0.05–0.21)	−4.83 (−5.20 to −4.46)
Republic of Korea	0.52 (0.09–1.14)	0.04 (0.02–0.11)	−9.44 (−10.11 to −8.76)

ASMR = age-standardized mortality rate, CI = confidence interval, DPR = Democratic People’s Republic, EAPCs = estimated annual percentage changes, UI = uncertainty interval.

**Table 3:** Age-standardized DALYs rate (ASDR) of upper respiratory infections and its temporal trends from 1990 to 2023.

Country	ASDR per 100,000 population (95% UI)		EAPCs (95% CI)
	1990	2023	
Global	110.64 (64.44–178.78)	83.94 (51.02–139.97)	−0.92 (−0.98 to −0.86)
China	128.13 (54.89–202.35)	53.25 (33.32–78.51)	−3.00 (−3.29 to −2.71)
DPR of Korea	70.45 (43.99–120.31)	57.08 (31.88–113.63)	−0.63 (−0.68 to −0.58)
Japan	99.57 (65.46–144.92)	88.52 (55.12–132.45)	−0.32 (−0.36 to −0.27)
Mongolia	91.07 (43.10–170.94)	45.59 (29.91–66.49)	−1.88 (−2.01 to −1.74)
Republic of Korea	94.53 (63.28–136.85)	83.06 (52.08–124.83)	−0.42 (−0.47 to −0.37)

ASDR = age-standardized DALYs rate, CI = confidence interval, DALYs = disability-adjusted life years, DPR = Democratic People’s Republic, EAPCs = estimated annual percentage changes, UI = uncertainty interval.

### 3.2. Segmented regression analysis of ASIR trends

**Figure 2.**
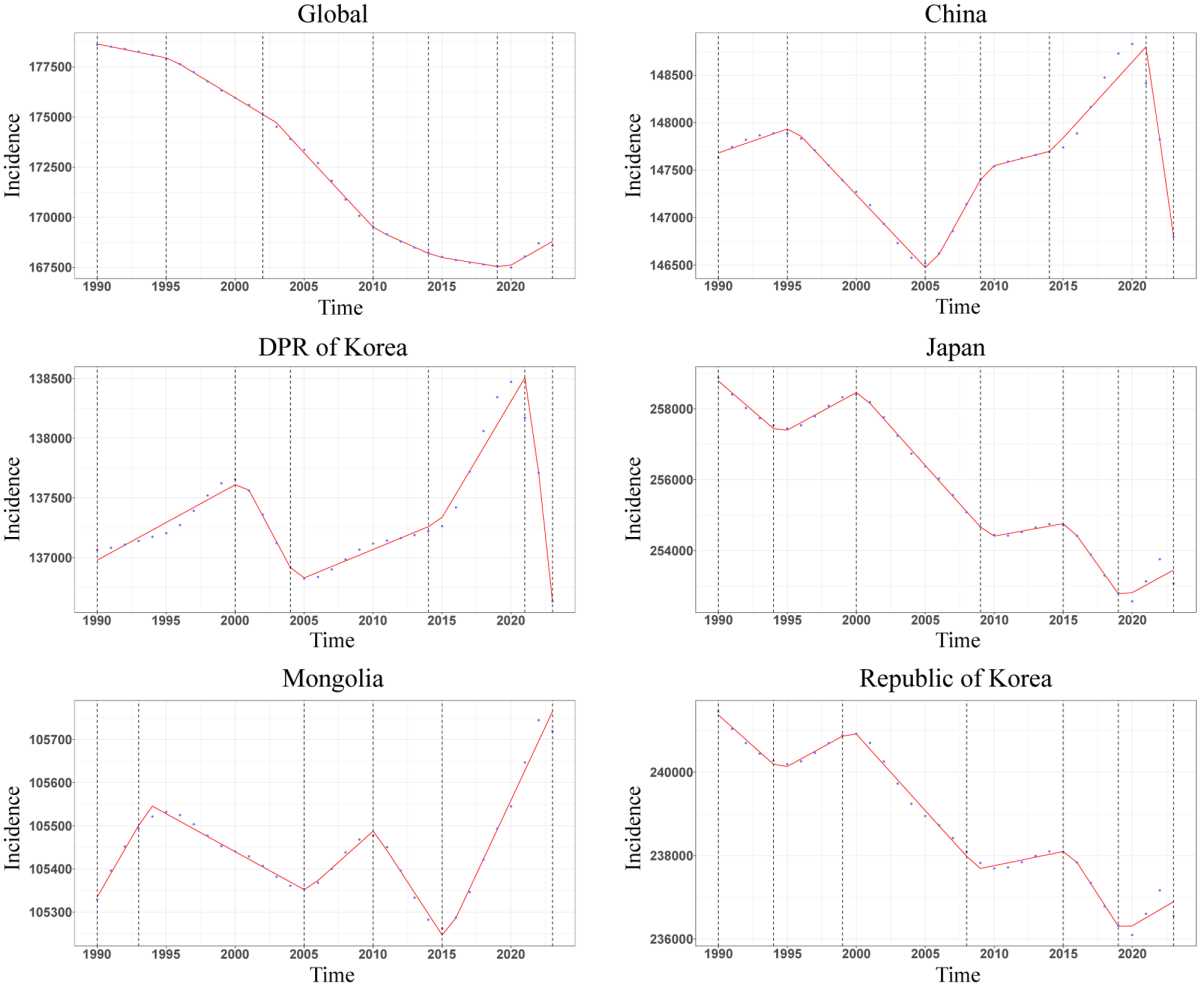
Joinpoint regression of the age-standardized incidence rate (ASIR, per 100,000) of upper respiratory infections among children and adolescents, 1990 to 2023, globally and in 5 East Asian countries. ASIR = age-standardized incidence rate, DPR = Democratic People’s Republic.

Segmented regression revealed substantial nonlinearity in ASIR trajectories that the single EAPC could not capture, with pandemic-period segments (2019–2023) explicitly quantified to account for COVID-19 disruptions (Fig. 2). Globally, ASIR declined through 5 successive segments from 1990 to 2019, with the steepest reduction during 2002 to 2010 (APC = −0.43%, 95% CI: −0.45 to − 0.41), followed by a significant rebound in 2019 to 2023 (APC = +0.23%, 95% CI: 0.17–0.30). In China, after a decade of decline in 1995 to 2005 (APC = −0.10%), ASIR rose during 2005 to 2009 (APC = +0.18%) and 2014 to 2021 (APC = +0.11%), before dropping sharply in 2021 to 2023 (APC = −0.69%, 95% CI: −0.91 to − 0.47). The DPR of Korea showed a similar late-period pattern, with an increase in 2014 to 2021 (APC = +0.14%) and a marked decline in 2021 to 2023 (APC = −0.78%, 95% CI: −0.99 to − 0.57). Japan and the Republic of Korea both exhibited alternating declines and rises, with renewed upturns in 2019 to 2023 (APC = +0.08% for both). Mongolia displayed the most fluctuating trajectory, ending with a sustained rise during 2015 to 2023 (APC = +0.06%, 95% CI: 0.06–0.07). These segment-specific shifts indicate clear COVID-19 – related perturbations and post-pandemic rebound effects across the region.

### 3.3. Age- and sex-specific distribution of URI burden among children and adolescents

**Figure 3.**
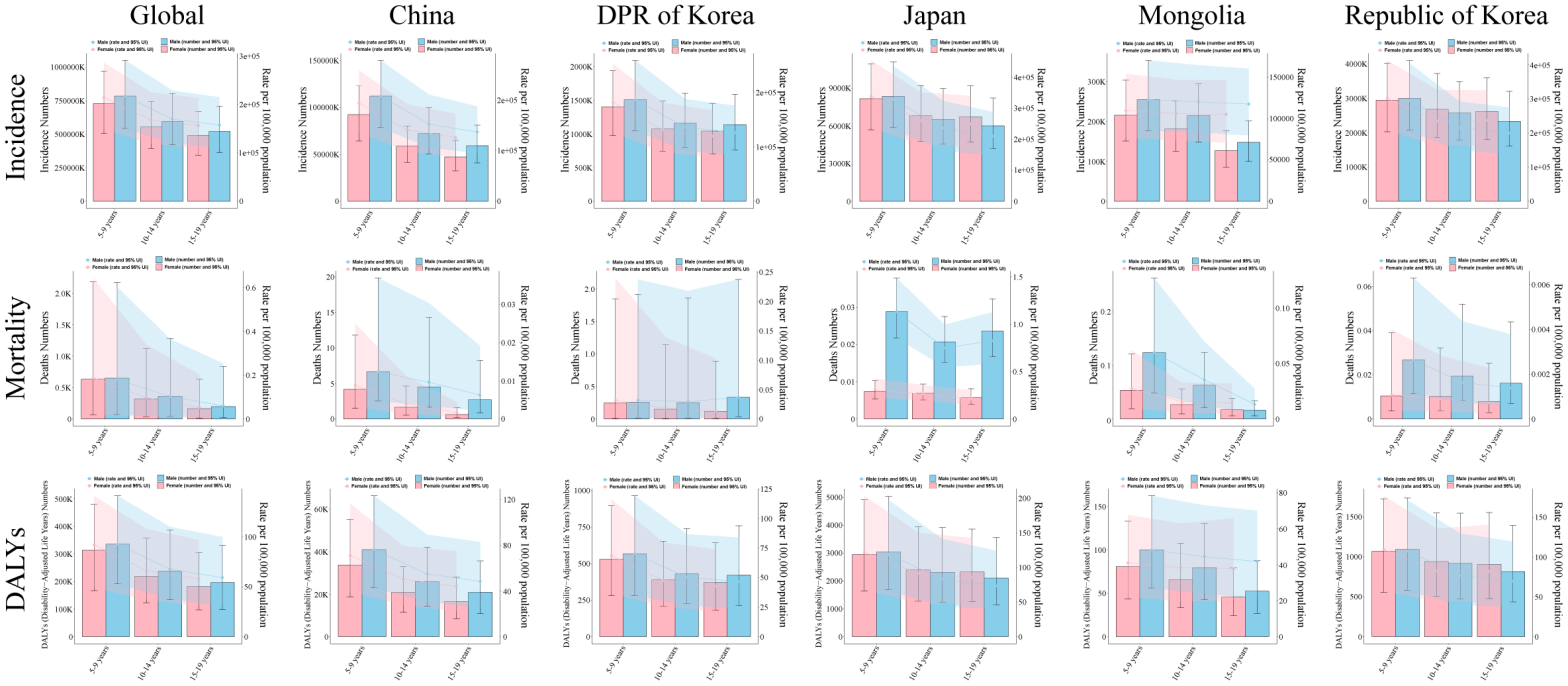
Sex- and age-specific burden of upper respiratory infections among children and adolescents globally and in 5 East Asian countries in 2023. Each panel displays case numbers (bars, left *y*-axis) and rates per 100,000 population (shaded areas, right *y*-axis) for males (blue) and females (pink), stratified by age group (5–9, 10–14, and 15–19 years). ASDR = age-standardized DALYs rate, DPR = Democratic People’s Republic.

In 2023, the incidence, mortality, and DALYs rates of URI declined with increasing age across all regions, with the 5- to 9-year group bearing the greatest burden (Fig. 3). For incidence, case numbers were slightly higher in males, though sex differences in rates were minimal. Japan recorded the highest incidence rates (males: 328,044 per 100,000; females: 338,303 per 100,000 in the 5- to 9-year group), while Mongolia had the lowest (males aged 5–9 years: 123,265 per 100,000). Notably, females exceeded males in incidence rates across all 3 age groups in Japan, and in the 10 to 14 and 15- to 19-year groups in the Republic of Korea. URI-related mortality was extremely low across all regions. In China, male mortality consistently exceeded female mortality, with the gap widening with age (5–9 years: 0.012 vs 0.009 per 100,000; 15 to 19 years: 0.006 vs 0.002 per 100,000), while rates in Japan and the Republic of Korea approached zero. DALYs rates followed a similar age-declining pattern. Japan had the highest rates among East Asian countries (5–9 years: males 119.08, females 122.34 per 100,000) and Mongolia the lowest (males 48.32, females 41.10 per 100,000). Males carried a slightly higher DALYs burden than females across most age groups and regions.

### 3.4. Relationship between SDI and DALYs rate

**Figure 4.**
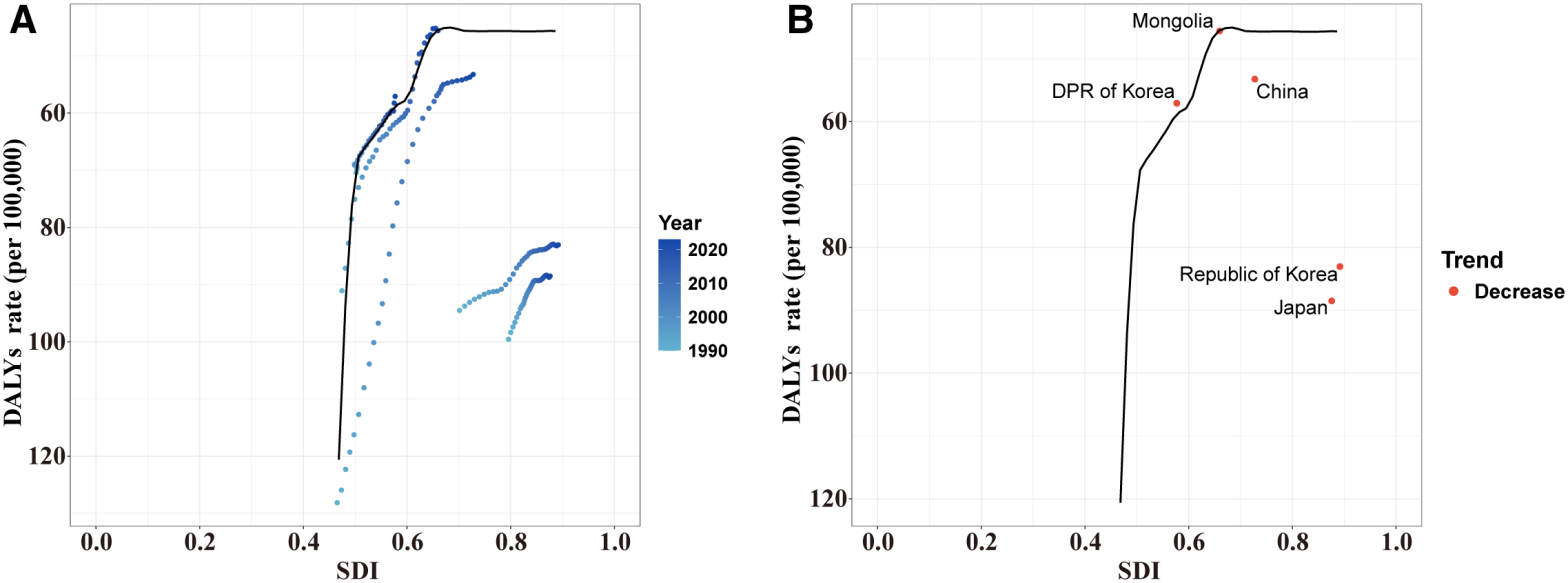
SDI and age-standardized DALYs rate (ASDR, per 100,000) of URI among children and adolescents globally and in 5 East Asian countries. SDI indicates socioeconomic development, and ASDR represents age-adjusted disease burden. (A) Displays 1990 to 2023 annual SDI-ASDR trajectories; the black curve is the global optimal frontier. Data points above the curve mean excess disease burden. (B) Presents 2023 frontier gaps (eff_diff). Japan and the Republic of Korea showed prominent excess URI burden despite advanced socioeconomic conditions, possibly due to high healthcare utilization, dense urbanization, and popular daycare use. ASDR = age-standardized DALYs rate, DALYs = disability-adjusted life years, SDI = socio-demographic index, URI = upper respiratory infection.

As SDI increased from 1990 to 2023, ASDR declined across all 5 East Asian countries, consistent with the globally expected pattern (Fig. 4A). China showed the greatest SDI gain (0.465 → 0.728) and the largest ASDR reduction (128.13 → 53.25 per 100,000), while the DPR of Korea had the smallest SDI increase (0.502 → 0.577). Comparison with expected frontier values revealed notable heterogeneity (Fig. 4B). The DPR of Korea fell on the frontier (eff_diff = 0), and China and Mongolia were close to it (eff_diff = 7.83 and 0.28, respectively), suggesting their URI burden is commensurate with their socioeconomic development. In contrast, Japan (eff_diff = 43.09) and the Republic of Korea (eff_diff = 37.31) had actual ASDR values substantially exceeding their expected frontier levels, which may reflect either genuine excess burden or more complete case ascertainment and reporting in these high-surveillance countries.

### 3.5. Decomposition analysis of changes in DALYs

**Figure 5.**
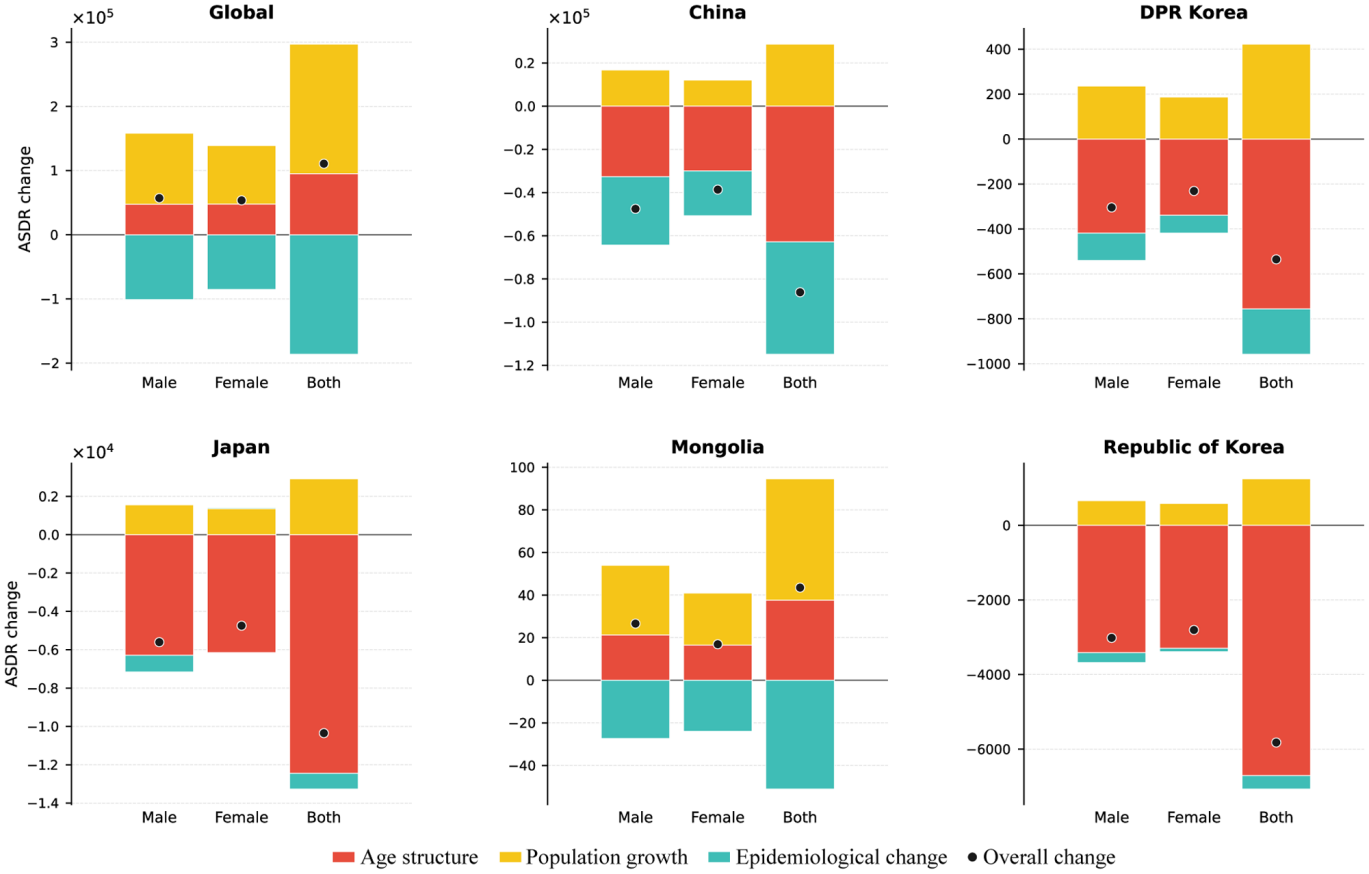
Decomposition analysis of changes in age-standardized DALYs rate (ASDR) of upper respiratory infections among children and adolescents from 1990 to 2023, stratified by sex and country. ASDR = age-standardized DALYs rate, DALYs = disability-adjusted life years.

Globally, absolute DALYs showed a net increase (+110,722), driven by population growth (+201,891) and age structure change (+95,183; i.e., shifts in age composition within the 5- to 19-year population rather than conventional population aging), partially offset by epidemiological improvement (−186,352) (Fig. 5). Among the 5 East Asian countries, Absolute DALYs declined in all except Mongolia. China recorded the largest reduction (−86,145), driven by age structure change (−62,776) and epidemiological change (−52,108). Japan (−10,352) and the Republic of Korea (−5,823) showed declines predominantly attributable to age structure change, while the DPR of Korea had the smallest decrease (−535). In contrast, Mongolia exhibited a slight net increase (+43), as population growth and age structure change outweighed epidemiological improvement. The magnitude of change was generally greater in males than females. Notably, the epidemiological change contribution was positive in Japanese females (+49.81) but negative in males (−871.42), suggesting divergent epidemiological trends between sexes in Japan.

### 3.6. APC Analysis

**Figure 6.**
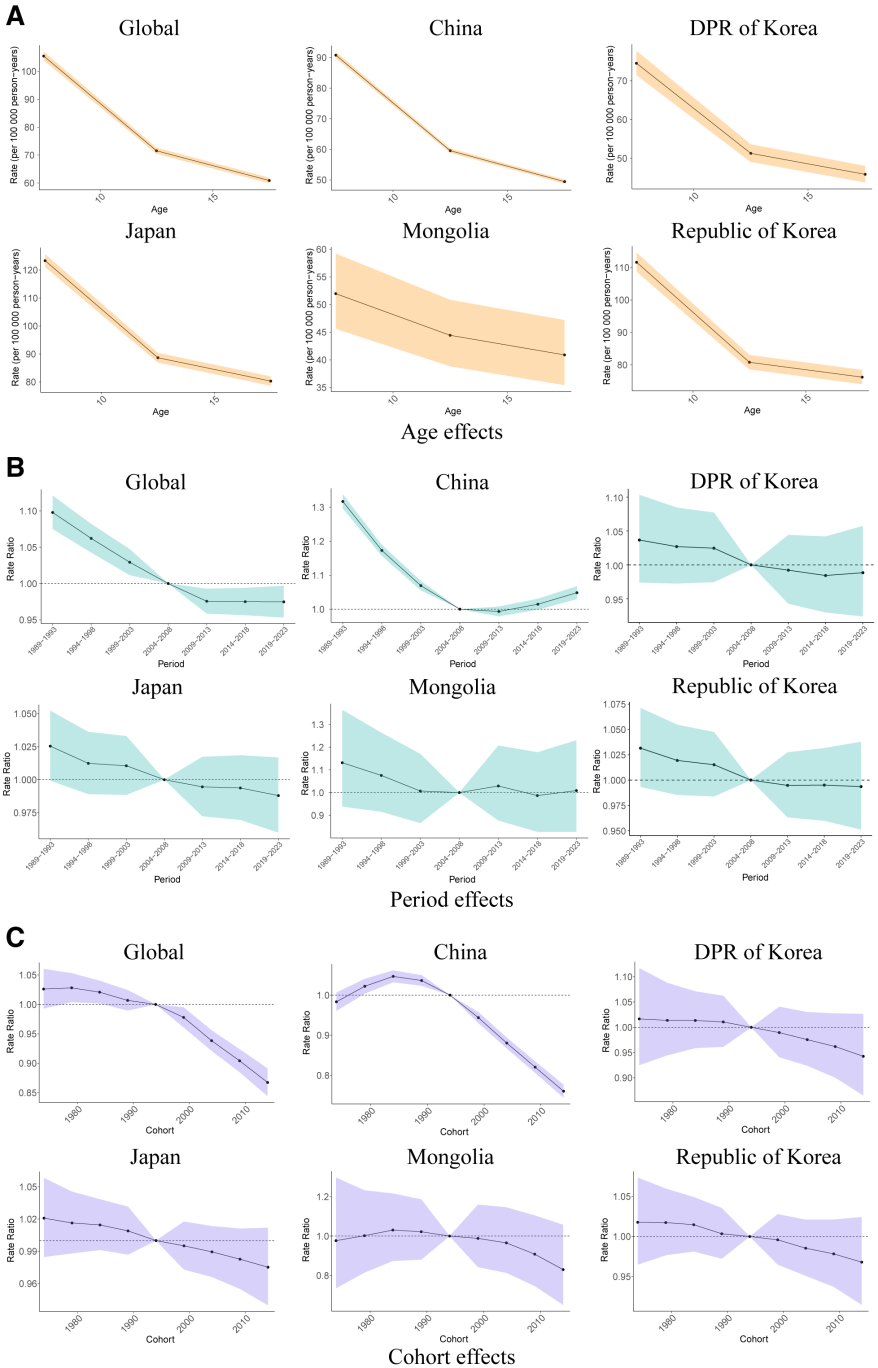
Age-period-cohort (APC) analysis of DALYs rates of upper respiratory infections among children and adolescents globally and in 5 East Asian countries. (A) Age effects expressed as rates per 100,000 person-years. (B) Period effects expressed as rate ratios relative to the reference period. (C) Cohort effects expressed as rate ratios relative to the reference birth cohort. Shaded areas represent 95% confidence intervals (CI). DALYs = disability-adjusted life years.

Regarding age effects, DALYs rates of URI among children and adolescents globally and across the 5 East Asian countries exhibited a marked age-dependent pattern, with the 5- to 9-year age group bearing the greatest burden and rates declining rapidly with increasing age (Fig. 6A). Japan recorded the highest rate in the 5- to 9-year group (123.35 per 100,000) and Mongolia the lowest (51.98 per 100,000); by the 15- to 19-year group, rates had declined substantially in all regions (Fig. 6A). Regarding period effects, the relative risk associated with calendar period showed a sustained downward trend from the early 1990s onward globally and in most countries, suggesting a beneficial impact of improvements in public health infrastructure and healthcare quality on URI burden (Fig. 6B). Notably, China exhibited a slight upturn in period risk during the most recent interval (2019–2023), a pattern that diverged from the trends observed in other countries. Regarding cohort effects, the relative risk for more recently born cohorts was lower than that of earlier cohorts across all regions, indicating a generational improvement in URI disease burden (Fig. 6C). Globally, the rate ratio for the 2012 to 2016 birth cohort declined to 0.867. The cohort effect was particularly pronounced in China, reflecting the long-term cumulative benefits of economic development, nutritional improvement, and expanded immunization programs.

### 3.7. Projected trends in URI DALYs burden through 2035

**Figure 7.**
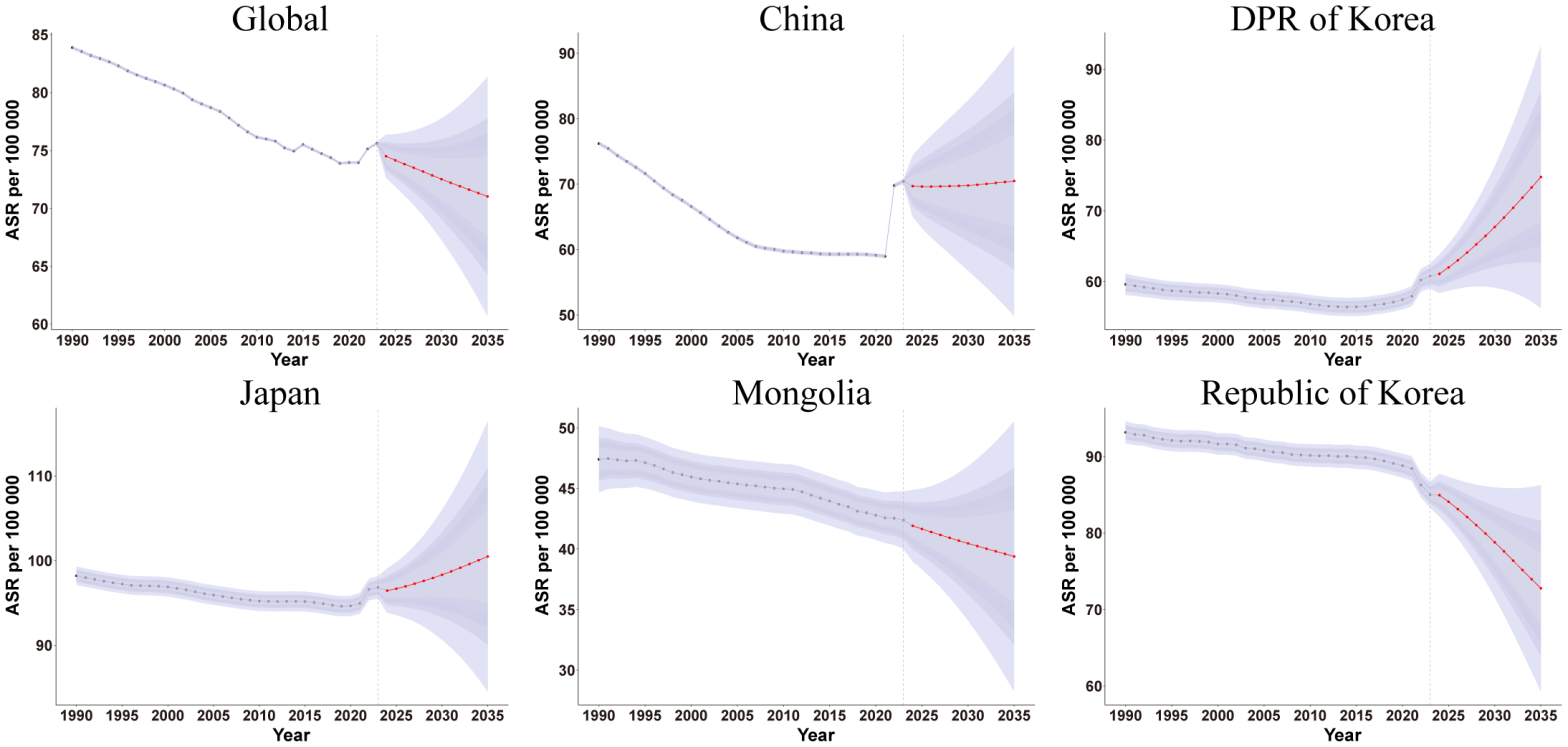
Projected trends in age-standardized DALYs rate (ASR per 100,000) of upper respiratory infections among children and adolescents from 1990 to 2035, globally and in 5 East Asian countries. Dotted lines represent observed trends from 1990 to 2023; red dashed lines indicate predicted trends from 2024 to 2035; shaded areas denote 95% prediction intervals estimated by the Bayesian age-period-cohort (BAPC) model. ASR = age-standardized rates, DALYs = disability-adjusted life years.

The BAPC model projected divergent future trajectories across the 6 regions (Fig. 7). Globally, the ASDR declined from 110.64 per 100,000 in 1990 to 73.90 per 100,000 in 2019, with a brief upturn to 83.94 per 100,000 in 2023, before resuming decline to a projected 71.03 (95% PI: 60.71–81.36) per 100,000 by 2035. In China, the ASDR declined to 53.25 per 100,000 in 2023 after pandemic-related fluctuations; however, BAPC projections suggest a subsequent rebound followed by a plateau at 70.47 (95% PI: 49.83–91.10) per 100,000 through 2035, reflecting persistent post-pandemic burden. The Republic of Korea showed a continued decline from 83.06 in 2023 to 72.80 (95% PI: 59.30–86.29) per 100,000 by 2035. Japan and the DPR of Korea were projected to show increasing trends, reaching 100.49 (95% PI: 84.56–116.43) and 74.79 (95% PI: 56.30–93.28) per 100,000 by 2035, respectively. Mongolia showed a modest decline to 39.38 (95% PI: 28.23–50.53) per 100,000 by 2035.

## 4. Discussion

The geographic distribution of URI disease burden across East Asian countries exhibited substantial heterogeneity. In 2023, Japan recorded the highest ASIR and Mongolia the lowest, a pattern closely linked to inter-country differences in healthcare accessibility, disease surveillance capacity, and reporting completeness.^[5]^ Notably, Japan and the Republic of Korea – the most socioeconomically advanced countries in the region – paradoxically ranked highest in both ASIR and ASDR, which may in part reflect their more comprehensive disease diagnosis and registration systems, enabling more complete identification and reporting of URI cases.^[21]^ By contrast, the ASIR in China and the DPR of Korea fell below the global average; given the persistent regional disparities in healthcare resource allocation and primary care capacity in these countries, the true burden of URI may be underestimated.

Between 1990 and 2023, ASMR declined significantly across all East Asian countries, with China (EAPC = −10.97) and Japan (EAPC = −10.35) recording the most pronounced reductions, consistent with the sustained investments made during this period in child healthcare, vaccine coverage, and public health infrastructure.^[22,23]^ The particularly steep decline in China is largely attributable to rapid economic development, widespread improvements in nutritional status, and progressively enhanced access to essential healthcare services for children over the past 3 decades.^[24]^ Nevertheless, disruptions to healthcare-seeking behavior and the strain on medical resources during the COVID-19 pandemic may have transiently perturbed mortality trends in certain regions, a factor that warrants careful consideration when interpreting recent data.^[25]^ Joinpoint regression further revealed that the single EAPC masked substantial temporal heterogeneity in ASIR trends. Globally, incidence declined most steeply during 2002 to 2010 (APC = −0.43%) but rebounded significantly in 2019 to 2023 (APC = +0.23%), reflecting post-pandemic resurgence of respiratory infections following relaxation of non-pharmaceutical interventions. In China, after alternating rises and declines, ASIR dropped sharply in 2021 to 2023 (APC = −0.69%), likely driven by intensified public health measures during the late pandemic period. Conversely, Japan and the Republic of Korea both exhibited renewed upward trends in 2019 to 2023, underscoring the region’s vulnerability to post-pandemic URI rebound despite high levels of socioeconomic development.

The URI disease burden exhibited a clear age-declining pattern, with the 5- to 9-year age group carrying the greatest burden, and males showing a slightly higher DALYs burden than females across all regions. Preschool and early school-age children are the most susceptible population for URI, owing to their immature immune systems and frequent close contact in congregate settings.^[26]^ As immunity matures with age, the incidence and severity of URIs decline, reflecting progressive immune development. Consistent with previous evidence, males tend to exhibit a slightly higher burden of respiratory infections. Peer et al^[27]^ 2025 reported higher incidence rates among males, potentially due to differences in immune response, behavioral exposure, and hygiene practices. Of note, females aged 10 to 14 and 15 to 19 years in Japan and the Republic of Korea exceeded their male counterparts in URI incidence rates, which may be related to increased participation in extracurricular activities, higher frequency of social interaction, and greater healthcare-seeking propensity among adolescent females; the underlying mechanisms warrant further investigation.

SDI analysis revealed an important finding: the observed ASDR in Japan (eff_diff = 43.09) and the Republic of Korea (eff_diff = 37.31) substantially exceeded the expected levels for their respective SDI values. Notably, mortality rates in these countries are extremely low (0.02–0.04 per 100,000), suggesting this pattern may largely reflect superior healthcare accessibility, diagnostic intensity, and more complete disease reporting rather than true excess morbidity. High-density urbanization and frequent healthcare utilization may also contribute, but the extent to which observed burden differences represent surveillance quality versus genuine epidemiological variation remains difficult to disentangle.^[28]^ By contrast, studies of tracheal, bronchus, and lung cancer indicate that high-SDI countries tend to achieve burden levels closer to the expected frontier, reflecting more effective alignment between socioeconomic development and health outcomes. Potential explanations include the high-density interpersonal contact associated with intensive urbanization,^[29]^ elevated URI diagnosis rates driven by high healthcare utilization and easy access to outpatient care,^[30]^ and increased exposure in congregate childcare settings linked to rising female labor force participation and expanded daycare enrollment in Japan and the Republic of Korea. Children attending daycare have consistently been shown to experience higher rates of respiratory infections than those cared for at home because of intensified close contact among susceptible individuals.^[31]^ These findings suggest that economic growth and healthcare investment alone are insufficient to automatically reduce URI burden, and that targeted interventions addressing urbanization and demographic risk factors are also required.

Decomposition analysis indicated that population growth and changes in age composition were the primary drivers of increasing absolute URI DALYs at the global level, while epidemiological improvement served as a significant offsetting force. It should be noted that the “aging” effect in this context refers specifically to shifts in the age composition within the study population (5–19 years) – namely, a structural redistribution from younger (5–9 years) toward older (15–19 years) age groups – rather than population aging in the conventional sense.^[32]^ In China, the substantial decline in DALYs was jointly driven by changes in age composition and epidemiological improvement, whereas Mongolia exhibited a net increase, as the effect of population growth outweighed epidemiological gains, highlighting the considerable URI burden pressure this country faces in the context of continued growth in its child population.

APC analysis demonstrated that more recently born cohorts carried a lower URI disease risk than earlier cohorts, suggesting that generational health improvements are broadly evident across East Asia, likely reflecting the long-term cumulative benefits of rising nutritional levels, expanded vaccine coverage, and improved living conditions.^[28,33]^ However, the modest upturn in the period effect observed in China during 2019 to 2023 warrants attention and may be attributable to a post-pandemic “immunity debt” phenomenon. While plausible, this interpretation remains speculative in the absence of pathogen-specific surveillance data, and reduced exposure to common respiratory pathogens during COVID-19-related non-pharmaceutical interventions may have led to an accumulation of susceptible individuals, resulting in a transient surge in respiratory infections following the relaxation of these measures, as described by Yang et al^[34]^ BAPC projections indicate that global ASDR and those of Mongolia and the Republic of Korea will continue to decline through 2035, while Japan and DPR of Korea are projected to show increasing trends, and China is expected to plateau. Japan’s rising disease burden and above-benchmark age-standardized death rate call for better prevention. Most URIs in this age group are mild, causing outpatient visits, school/work absences, and antibiotic use instead of severe outcomes. High incidence strains primary care and family productivity.^[1]^ The DPR of Korea’s projected prevalence increase likely results from weak surveillance and primary care, while limited local epidemiological data hinders in-depth analysis.^[35]^ China’s plateauing ASDR, echoing the post-pandemic period effect rebound, signals the need for continued vigilance against short-term burden resurgence. Collectively, these projections underscore the necessity of developing differentiated, forward-looking URI prevention and control strategies tailored to the specific circumstances of each country.

The findings of this study carry direct policy implications for pediatric respiratory infection control in East Asia. For high-burden, high-SDI countries (Japan and Republic of Korea), despite excellent healthcare infrastructure, interventions should prioritize non-pharmaceutical measures: promoting hand hygiene and respiratory etiquette in schools, optimizing ventilation in crowded settings, and implementing targeted vaccination campaigns for influenza and pneumococcal disease among high-risk pediatric groups.^[36]^ In contrast, for countries with improving but heterogeneous healthcare systems (China and Mongolia), strengthening primary care capacity for early URI detection and management, expanding rural healthcare access, and addressing urban-rural disparities in vaccine coverage remain paramount. The post-pandemic rebound observed across the region underscores the need for maintaining core public health capacities even during inter-pandemic periods. Compared to other regions, East Asia’s URI burden patterns resemble those in high-density urban areas of Europe and North America, where despite advanced healthcare systems, transmission-intensive settings sustain elevated incidence; however, East Asian mortality rates are notably lower, reflecting superior access to pediatric care.^[37]^

This study has several limitations. First, GBD estimates are based on statistical modeling that incorporates primary data of varying quality and completeness. For countries with robust surveillance systems (e.g., Japan and Republic of Korea), estimates likely reflect actual disease burden; however, for DPR of Korea and Mongolia, where primary epidemiological data are sparse, the observed patterns may partly reflect modeling assumptions and data availability rather than true epidemiological differences. Country-specific comparisons should therefore be interpreted with appropriate caution. Second, the explanatory hypotheses including healthcare-seeking behavior, activity patterns, urbanization, and immunity debt remain speculative without empirical validation, which requires individual or pathogen-specific data. Third, though the 2020 to 2023 pandemic brought reporting biases, behavioral shifts, and intervention impacts, we did not perform separate pre- and post-pandemic trend analyses. While joinpoint regression identified pandemic-related inflection points, residual confounding cannot be fully ruled out. Finally, the BAPC model relies on extrapolation from historical trends and cannot account for unpredictable future events such as major public health emergencies, climate change, or the emergence of novel pathogens; projections should therefore be interpreted with appropriate caution.

## 5. Conclusion

From 1990 to 2023, the overall URI disease burden among children and adolescents in East Asia declined, yet marked heterogeneity was observed across countries. Japan and the Republic of Korea continued to bear a disproportionately high burden relative to their level of socioeconomic development, while BAPC projections indicate that the burden in Japan and DPR of Korea will rise further in the coming years. These findings highlight the need for East Asian countries to develop targeted URI prevention and control strategies tailored to their specific demographic structures and healthcare system capacities in order to address the ongoing disease burden challenges ahead.

## Acknowledgments

The authors acknowledge the GBD 2023 collaborators and Institute for Health Metrics and Evaluation, University of Washington, for making the data publicly available. The views expressed in this study are solely those of the authors and do not necessarily represent those of Institute for Health Metrics and Evaluation or its funding agencies.

## Author contributions

**Conceptualization:** Dong Chen, Botong Sun.

**Data curation:** Dong Chen, Yujie Qian.

**Formal analysis:** Dong Chen, Yujie Qian.

**Investigation:** Dong Chen, Jing Ma, Lei Zhang.

**Methodology:** Botong Sun, Dan Qian, Zhi Xu.

**Software:** Dong Chen, Zhi Xu.

**Supervision:** Zhi Xu.

**Writing – original draft:** Dong Chen, Botong Sun.

**Writing – review & editing:** Zhi Xu.
